# Parameterization, algorithmic modeling, and fluid–structure interaction analysis for generative design of transcatheter aortic valves

**DOI:** 10.1007/s00366-024-01973-5

**Published:** 2024-06-27

**Authors:** Xianyu George Pan, Ashton M. Corpuz, Manoj R. Rajanna, Emily L. Johnson

**Affiliations:** 1 Department of Aerospace and Mechanical Engineering, University of Notre Dame, Notre Dame, IN USA; 2 Department of Mechanical Engineering, Iowa State University, Ames, IA USA; 3 Global Engineering and Materials, Inc., Princeton, NJ USA

**Keywords:** Parametric modeling, Transcatheter heart valve, Fluid–structure interaction, Immersogeometric analysis, TAVR

## Abstract

Heart valves play a critical role in maintaining proper cardiovascular function in the human heart; however, valve diseases can lead to improper valvular function and reduced cardiovascular performance. Depending on the extent and severity of the valvular disease, replacement operations are often required to ensure that the heart continues to operate properly in the cardiac system. Transcatheter aortic valve replacement (TAVR) procedures have recently emerged as a promising alternative to surgical replacement approaches because the percutaneous methods used in these implant operations are significantly less invasive than open heart surgery. Despite the advantages of transcatheter devices, the precise deployment, proper valve sizing, and stable anchoring required to securely place these valves in the aorta remain challenging even in successful TAVR procedures. This work proposes a parametric modeling approach for transcatheter heart valves (THVs) that enables flexible valvular development and sizing to effectively generate existing and novel valve designs. This study showcases two THV configurations that are analyzed using an immersogeometric fluid–structure interaction (IMGA FSI) framework to demonstrate the influence of geometric changes on THV performance. The proposed modeling framework illustrates the impact of these features on THV behavior and indicates the effectiveness of parametric modeling approaches for enhancing THV performance and efficacy in the future.

## Introduction

The valves in the human heart are crucial components in the proper function of the overall cardiovascular system. When operating correctly, these valves regulate blood flow in the heart, ensuring sufficient circulation throughout the body. On the left side of the heart, the aortic valve is responsible for directing blood from the left ventricle to the aorta while preventing any backward flow [1]. However, calcification and other valvular diseases can impair the aortic valve, leading to regurgitation or stenosis. Such conditions are characterized by improper blood flow regulation and inadequate flow properties, which can induce additional stress on the cardiovascular system as the heart compensates to maintain an adequate blood supply to the body [2]. In cases of severe aortic valve disease, replacement is the recommended and often necessary treatment for restoring proper functionality to the cardiovascular system [3, 4].

Traditional treatment options for severe valvular disease typically involve open-heart surgery to replace the affected valve with a prosthetic device [3]. While these surgical procedures have relatively high success rates [5], the more invasive nature of such surgeries can lead to considerable morbidity and death, particularly in older or high-risk patients. Transcatheter aortic valve replacement (TAVR) provides a minimally invasive intervention option that leverages percutaneous methods to replace the native valve with a bioprosthetic transcatheter heart valve (THV). Such procedures also offer several advantages over surgical replacement operations, including the reduced risk of infection, shorter procedure time, shorter hospital stays, faster recovery times, and improved symptoms, quality of life, and life expectancy [6, 7].

Although transcatheter replacement approaches have many advantages, a core challenge with such devices is ensuring the secure placement of the prosthesis in a suitable position in the aorta. Successful implant procedures require accurate deployment, proper valve sizing, and stable anchoring of the device, which rely heavily on the design and relative dimensions of the valve implant [8, 9]. Failure to meet these criteria can result in other complications, including coronary obstruction, annular rupture, paravalvular regurgitation, poor device fixation, and valve migration, which become more likely if the valve is improperly sized for a particular patient [10–13].

In an effort to examine these challenges, recent work investigating the mechanics and hemodynamics of bioprosthetic devices has centered on computational modeling approaches, which enable efficient simulation and analysis. The THV system has been studied using various simulation approaches, including structural mechanics simulations [14–19], computational fluid dynamics [20, 21], and fluid–structure interaction (FSI) [22–25]. Available evidence also indicates that specific geometric valve characteristics and parameters can significantly influence valve performance [26–29]. Several studies investigating the impact of different valvular parameters and dimensions have identified leaflet curvature in the belly region and at the lower attachment edge as significant parameters in the performance of bioprosthetic heart valves, particularly related to valvular stress during crimping and throughout the cardiac cycle [30–32].

**Fig. 1:**
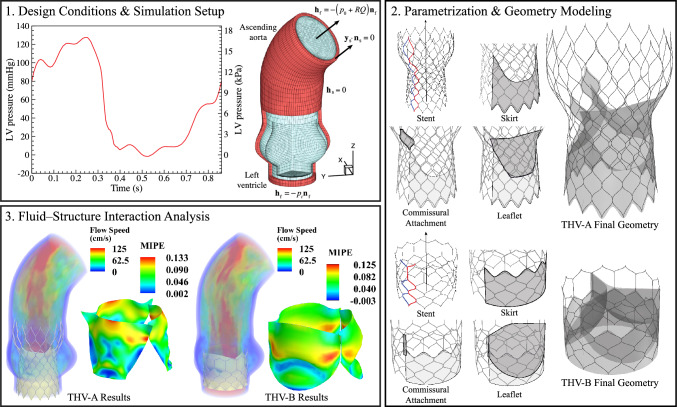
Overview of the design conditions and simulation setup, THV geometry modeling approaches, and FSI results at $t = 0.25$ s for each valve configuration demonstrated in this work

The present work proposes and details a versatile parametric approach that addresses some of the concerns and challenges associated with transcatheter heart valves by providing a flexible modeling framework for valvular design and device sizing and evaluation. The proposed modeling algorithms are intended to offer a method to more comprehensively investigate and understand the impact of the geometric features of transcatheter devices. This framework includes procedures to describe and algorithmically model the THV stent, skirt, and leaflets to enable the generative design of stented transcatheter valves. The effectiveness of this approach is demonstrated through the construction of two different valve configurations that are simulated using an immersogeometric fluid–structure interaction (IMGA FSI) framework [33, 34] to explore the behavior of these THVs in a physiologically realistic environment [25]. A schematic of the computational modeling and simulation approaches in this work is shown in Fig. 1. The parameterization and geometry modeling approaches developed in this work are first outlined in Sect. 2. The IMGA FSI simulation methods are then summarized in Sect. 3. The results and discussion of the two realistic valve configurations demonstrated in this work are presented in Sect. 4, and conclusions are summarized in Sect. 5.

## Parametric modeling of transcatheter heart valves

The proposed parametric modeling algorithms provide a comprehensive approach for generating numerous designs and configurations of transcatheter heart valves, including existing and novel designs. The following sections detail the parameters and geometry modeling algorithms that are developed to describe a generalized approach for defining the individual components of a transcatheter heart valve. These algorithms are demonstrated for two specific valve configurations, THV-A and THV-B, that closely approximate realistic valves: the Medtronic CoreValve$^{\textrm{TM}}$ Evolut$^{\textrm{TM}}$ R System [35, 36] and the Edwards SAPIEN 3 System [37, 38], respectively. To differentiate the geometries in the generation algorithms for each THV component, the following color conventions are adopted for the majority of the geometries presented in this section: red geometries at individual stages of the construction procedures are generally used to indicate an “active” or “current” geometry that is used in that step of the algorithm, blue geometries are generally used to indicate translation and additional dimensions or parameters, and black or grey geometries are generally used to indicate reference geometries and geometries that were used in a previous step of the construction procedure.

### Stent

The first THV component constructed in this procedure is the stent or frame of the valve. Just as the stent in an actual valve provides the overall structure for the THV geometry and supports the skirt and leaflets, the parameterized stent provides the basis for many other components in the THV model. Many of the stent parameters in this model serve as the source for the skirt and leaflet parameters that are subsequently defined, making the stent parameters crucial control factors in the construction of the THV geometry.

**Table 1:** THV-A stent parameters based on the Evolut R system dimensions [35, 36]

	$r_c$ (mm)	$\phi _c$ ($^{\circ }$)	$z_c$ (mm)
$P_1$	13.15	0	0.00
$P_2$	12.55	12	5.00
$P_3$	12.20	0	9.00
$P_4$	11.75	12	13.00
$P_5$	11.55	0	16.50
$P_6$	11.00	12	20.00
$P_7$	11.00	0	23.50
$P_8$	12.65	12	27.50
$P_9$	15.20	0	32.50
$P_{10}$	15.85	12	38.50
$P_{11}$	14.90	0	45.00

**Table 2:** THV-B stent parameters based on the SAPIEN 3 system dimensions [37, 38]

	$r_c$ (mm)	$\phi _c$ ($^{\circ }$)	$z_c$ (mm)
$P_1$	13.00	0	0.00
$P_2$	13.00	15	3.65
$P_3$	13.00	0	6.70
$P_4$	13.00	15	9.70
$P_5$	13.00	0	12.75
$P_6$	13.00	15	15.20
$P_7$	13.00	0	17.60
$P_8$	13.00	15	20.05

**Fig. 2:**
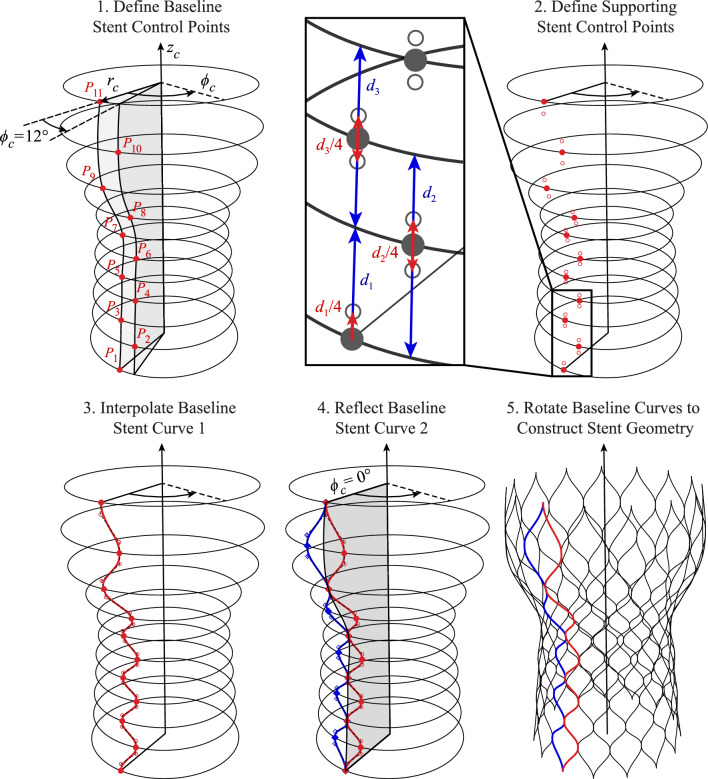
Parameters and construction procedure for the THV-A stent based on the proposed algorithmic modeling approach

**Fig. 3:**
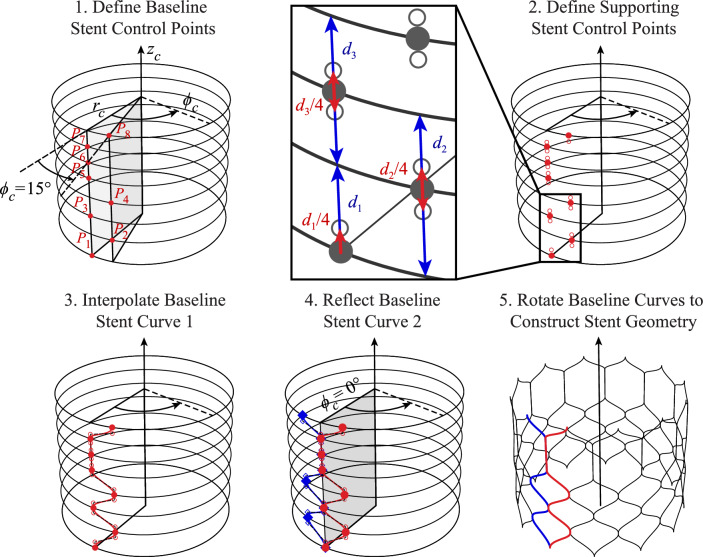
Parameters and construction procedure for the THV-B stent based on the proposed algorithmic modeling approach

The main basis for the THV stent is a set of points, described in cylindrical coordinates, that define the overall stent geometry. Since the overall frame is assumed to be radially symmetric, these cylindrical coordinates define the radius and height of each stent cross-section, $r_c$ and $z_c$, respectively, and the azimuthal angle, $\phi _c$, determines the number of stent sections that will be constructed. The number of points and the coordinates can both be modified to achieve many different valve configurations, as indicated by the coordinates in Tables 1 and 2 that will be used to construct geometries based on the Evolut R and SAPIEN 3 valves. As more points are added, additional cross-sections can be modeled. The procedure and set of parameters used to describe the THV stent are shown in Figs. 2 and 3.

As the first step in the geometry generation process for the stent, the defined set of baseline points is first constructed, as shown in Step 1 of Figs. 2 and 3 for the THV-A and THV-B valves, respectively. These points define the physical location where two stent wires will intersect in the final geometry; however, additional support points are necessary to maintain consistent curve tangents at the point where two curves are connected. For a cubic non-uniform rational B-spline (NURBS) curve, the local tangents at this connection point can be maintained by three collinear control points [25]: one baseline point and two supporting points. For the top and bottom connection points at the maximum and minimum $z_c$ locations, respectively, these supporting points are linearly offset by 25% of the distance between the adjacent vertical cross-sections above or below the connection point (e.g., $d_1$ in the inset of Figs. 2 and 3). For any interior connection points, the supporting points are linearly offset by 12.5% of the distance between the adjacent vertical cross-sections above and below the connection point (e.g., $d_2$ and $d_3$ in the inset of Figs. 2 and 3), which results in a consistent relative offset distance between all the support points that maintains a reasonably scaled curvature at the connection points. These distances can also be modified to achieve “smoother” or “sharper” attachments between the final stent curves. The definition of the support points for the stent is shown in Step 2 of Figs. 2 and 3. Once the baseline and support control points are defined, a single cubic NURBS curve can be constructed as the stent curve, as shown in Step 3 of Figs. 2 and 3. From this first curve, the second baseline curve can be constructed by reflecting the first curve across the vertical plane at $\phi _c = 0^{\circ }$ (Step 4 of Figs. 2 and 3). Because both curves were constructed consistently based on $\phi _c$ to achieve a symmetric distribution of sections around the stent geometry, this set of two curves can be rotated about the $z_c$-axis in increments of twice the maximum $\phi _c$ angle to achieve the final set of stent curves, as shown in Step 5 of Figs. 2 and 3.

When analyzing the stent frame, two control points that are overlapped in the undeformed configuration are treated as a single control point during the simulation. In any stent configuration, the three collinear control point pairs at each connection point are constrained to move together, so the physical location of any shared points will remain consistent throughout the deformation, ensuring the appropriate tangency at this location. This configuration also maintains the desired clamped condition between two stent wires and transfers the moment through the connection point [25].

**Fig. 4:**
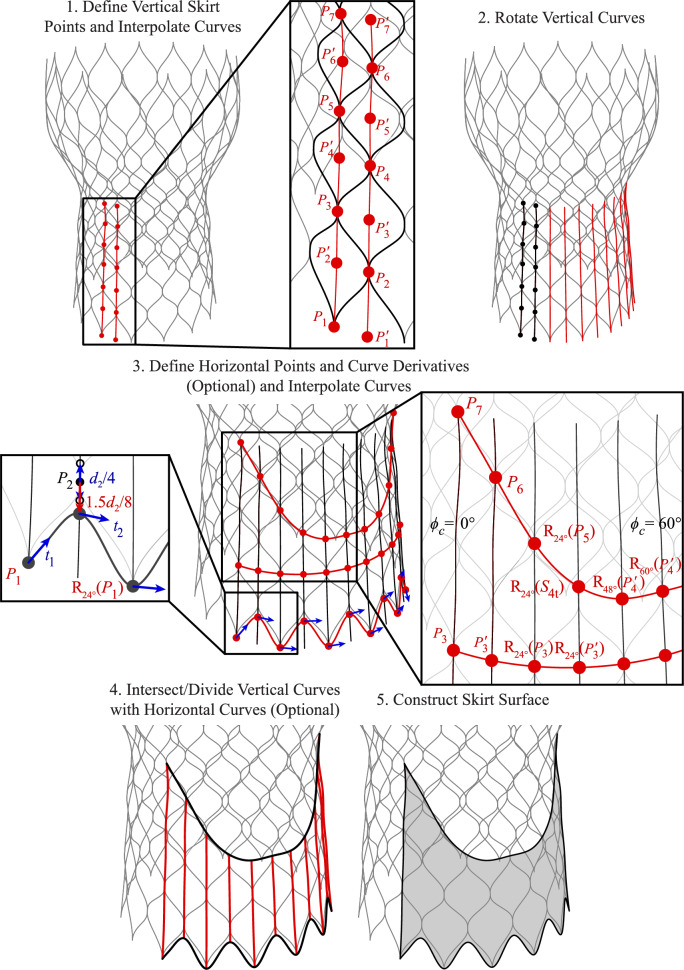
Parameters and construction procedure for the THV-A skirt based on the proposed algorithmic modeling approach

**Fig. 5:**
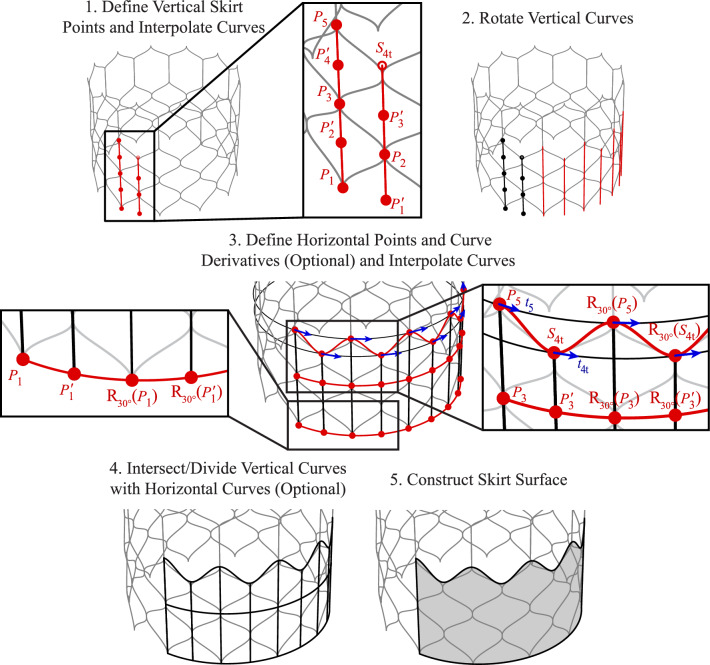
Parameters and construction procedure for the THV-B skirt based on the proposed algorithmic modeling approach

### Skirt

The next step of the geometry construction process focuses on the skirt, and the parameters for this procedure are shown in Figs. 4 and 5. The first geometries constructed are the two vertical curves that provide the baseline for the skirt. These curves are defined by interpolating a combination of the baseline stent control points (*P*), baseline stent control points with inverted $\phi$ coordinates (indicated as $P\,'$), and supporting points where relevant (indicated as $S_t$ or $S_b$ for the top or bottom support point, respectively), as shown in Step 1 of Figs. 4 and 5. Once these two vertical curves are constructed, they are rotated by consistent angles in increments of $\phi _c$ about the central $z_c$-axis to generate one-third of the skirt, as shown in Step 2 of Figs. 4 and 5. In Step 3 of Figs. 4 and 5, the horizontal skirt curves that define the top and bottom edges of the skirt are constructed based on another set of stent baseline control points, supporting stent control points, and a subset of rotated or translated baseline or supporting stent control points that approximate the skirt configuration for the desired THV geometry. Curve derivatives can also be incorporated for these horizontal curves if necessary to maintain consistent tangents at each point.

In Step 3 of Figs. 4 and 5 and future geometry construction steps, $\text {R}_{\phi _c}(\varvec{\cdot })$ indicates a geometry rotation by an angle of $\phi _c$ degrees about the $z_c$-axis (e.g., in Step 3 of Fig. 4, $R_{{24^{\circ }}}(P_{1})$ indicates that $P_1$ is rotated $24^{\circ }$ about the $z_c$-axis). Rotations about the $z_c$-axis are evaluated in increments of the maximum $\phi _c$-coordinates of the stent for each configuration. The geometry is also radially symmetric about the *z*_*c*_-axis, and the third of the geometry that is defined between $\phi _c = 0^{\circ }$ and $\phi _c = 120^{\circ }$ is symmetric across the vertical plane at $\phi _c = 60^{\circ }$. With these horizontal curves defining the top and bottom edge of the skirt, the vertical curves that were defined in Step 2 can be split, if necessary, into smaller sections based on the intersection points that are used to construct the top and bottom horizontal curves, as shown in Step 4 of Figs. 4 and 5. The skirt is then constructed as a Gordon surface from the set of vertical and horizontal cubic NURBS curves that describe the bidirectional curve network defining the surface, and the final geometry is shown in Step 5 of Figs. 4 and 5.

**Fig. 6:**
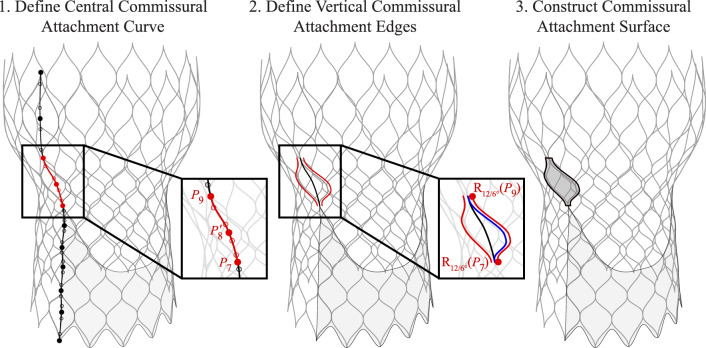
Parameters and construction procedure for the THV-A commissural attachments based on the proposed algorithmic modeling approach

**Fig. 7:**
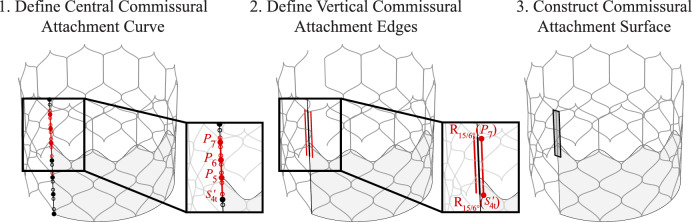
Parameters and construction procedure for the THV-A commissural attachments based on the proposed algorithmic modeling approach

### Commissural attachments

In this work, the surfaces that attach the upper portions of each leaflet to the stent are designated as the “commissural attachments.” These surfaces are critical structures that maintain the leaflet shape and, in practice, ensure that the leaflets can be sutured to the upper section of the stent. The construction procedure for the commissural attachments is shown in Figs. 6 and 7. The initial commissural attachment surface is constructed based on a section of the baseline stent curve. When defining these surfaces, a central curve at $\phi _c = 0^{\circ }$ is first defined based on a combination of the baseline stent control points, baseline stent control points with inverted $\phi$ coordinates, and supporting points where relevant, as shown in Step 1 of Figs. 6 and 7. Once the central curve is constructed, the two vertical edges of the commissural attachment are then defined, as shown in Step 2 of Figs. 6 and 7. For this step, a specified curve, either the central commissural curve or the section of the baseline stent curve with the same endpoints as the central commissural curve, is rotated about the central $z_c$-axis by $\phi _c/6$ for the maximum $\phi _c$ stent coordinate and is then reflected across the vertical plane at $\phi _c = 0^{\circ }$. These two edges and the central commissural curve define a set of three curves that are then lofted to create the commissural attachment surface shown in Step 3 of Figs. 6 and 7.

**Fig. 8:**
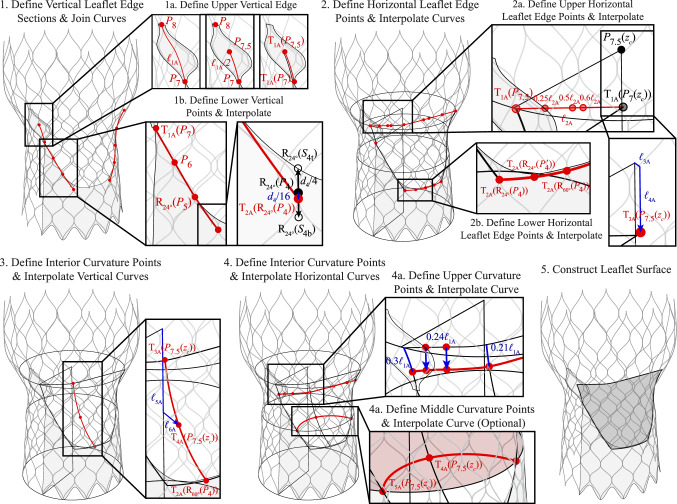
Parameters and construction procedure for the THV-A leaflets based on the proposed algorithmic modeling approach

**Fig. 9:**
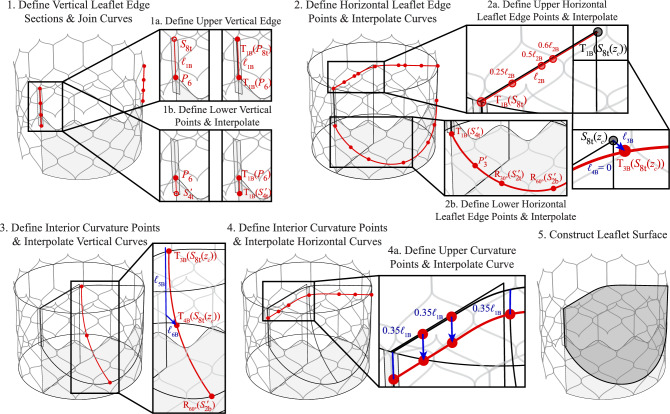
Parameters and construction procedure for the THV-B leaflets based on the proposed algorithmic modeling approach

### Leaflets

The leaflet parameterization is similar to a previous aortic valve parameterization that controls the leaflet attachment edges, free edge, and central curvature [29]. This general approach is adapted to work effectively with the THV configuration. The reference points for the vertical edges of the leaflets are first identified from the stent baseline points and the commissural attachment surfaces. As shown in Step 1 of Figs. 8 and 9, the leaflet edges are constructed from two generated curve sections that are joined at their common endpoints. Step 1a shows the process for constructing the upper portion of the vertical edge from the central commissural attachment edge. This central curve is first extracted and, if necessary, divided into subsections to account for any offset of the vertical edge of the leaflet from the top or bottom of the commissural attachment surface. Once the full or partial central curve is extracted, it can be translated along the tangential direction of the commissural surface normal to the vertical plane at $\phi _c = 0^{\circ }$. Geometry translation operations on points or curves are indicated by $T (\varvec{\cdot })$ (e.g., in Step 1a of Fig. 8, $T_{\text {1A}}(P_{7})$ indicates the first geometry translation operation for the THV-A geometry, which is performed on $P_7$). At this step, the curves are translated by 0.12 mm and 0.15 mm for the THV-A and THV-B valves, respectively. The lower portion of the vertical edge curve is constructed from several points that are extracted from a combination of the set of baseline stent control points, baseline stent control points with inverted $\phi$ coordinates, supporting points, and transformed points (rotations or translations) from the same set, as shown in Step 1b of Figs. 8 and 9. The generated vertical curve geometry is then reflected across the vertical plane at $\phi _c = 60^{\circ }$.

The next set of geometries generated in the leaflet construction procedure is the horizontal leaflet edge curves at the free edge and the bottom edge of the leaflet that is attached to the skirt, as shown in Step 2 of Figs. 8 and 9. The sides of the upper horizontal curve are constructed to maintain linear curve tangents along longer sections of the sides of the leaflet’s free edge and ensure that adjacent leaflets do not intersect in the initial geometry configuration. A set of linear points is evaluated on the line that joins the top point on the vertical leaflet edge curve and a point near the $z_c$ axis (Step 2a). The location of the point near the central axis varies based on the geometry being constructed. For a leaflet configuration in which the central point of the free edge is lower than the corners of the leaflet, as in the THV-A configuration, the other end of the generated line is selected at the $z_c$-coordinate of the bottom point of the upper vertical leaflet edge section, which is at the bottom of the commissural attachment surface. This point can be further offset in the vertical direction to consistently approximate particular leaflet shapes. For a leaflet configuration in which the central point of the free edge is at the same height as the corners of the leaflet, as in the THV-B configuration, the point near the $z_c$-axis is defined as the $z_c$-coordinate of the top point of the upper vertical leaflet edge section. In Figs. 8 and 9, the $z_c$-coordinate of a point, $P(0,0,z_c)$, is abbreviated as $P(z_c)$.

Once this line is defined, points are selected at specified increments along it to define the side portions of the top edge of the leaflet and maintain a straight edge in this region of the leaflets. Once this set of points is defined, all the points are reflected across the vertical plane at $\phi _c = 60^{\circ }$. The final point defined for the upper leaflet edge is the central point, which is offset from a point on the $z_c$ axis with the same $z_c$-coordinate as the center point at the top of the commissural attachment surface. The point is offset radially along the plane at $\phi _c = 60^{\circ }$ and then offset vertically, if necessary, to approximate the desired valvular configuration. The sets of linear points and the central point are then interpolated to generate the upper horizontal leaflet curve.

The lower horizontal leaflet attachment curve, shown in Step 2b of Figs. 8 and 9, is constructed from translated and rotated points from the baseline stent control points, baseline stent control points with inverted $\phi$ coordinates, and supporting points. These points are generated symmetrically across the vertical plane at $\phi _c = 60^{\circ }$ and interpolated.

Once the leaflet edges are generated, the central leaflet curvature can then be defined by several additional curves: one central interior vertical curve and at least one horizontal interior curve. Step 3 of Figs. 8 and 9 shows the process for generating the vertical curve. This geometry is interpolated from the central points on the leaflet free edge and bottom leaflet attachment edge as well as one central point that is defined similarly to the central point on the leaflet free edge by offsetting the same point vertically and radially along the plane at $\phi _c = 60^{\circ }$ to approximate the desired valvular curvature.

Step 4 of Figs. 8 and 9 shows the process for generating the interior horizontal curve(s). One upper curve is defined by offsetting and interpolating a subset of the points that define the free edge of the leaflet. The endpoints and central point are offset along the leaflet edge curves and central curve, respectively, by a factor of a selected length (the length of the upper vertical edge, as defined in Step 1a of Figs. 8 and 9), and two additional points from each side portion of the free edge curve are offset in the $-z_c$-direction by a factor of the same length. Additional horizontal curves can also be generated to control the leaflet curvature in the lower portions of the leaflet if desired. For the THV-A configuration shown in Fig. 8, one additional planar arc is generated using the central point from the vertical interior curve and the two points on the leaflet vertical edges with the same $z_c$-coordinate.

The final set of vertical and horizontal curves is then used to construct one valve leaflet as a Gordon surface, and the final leaflet geometry is shown in Step 5 of Figs. 8 and 9.

### Final geometry

Finally, to create all the surfaces for the full TAVR geometry, the skirt, leaflets, and commissural attachments are replicated and rotated about the $z_c$-axis to create the remaining two-thirds of the geometry, as shown in Fig. 1. The discretization of the surfaces and curves is also reconstructed and refined where necessary to obtain relatively uniform surface and curve discretizations that are analysis-suitable.

## Simulation framework

In this section, we summarize the main components of the immersogeometric FSI framework for modeling the THV system. For this model, the FSI problem includes the blood flow, the arterial wall, and the various components of the THV. For more detailed mathematical and implementation details, readers are referred to Wu et al. [25].

### Fluid–structure interaction problem

The THV and ascending aorta at time *t* are modeled as elastic structures occupying region $(\Omega _\text {s})_t$, coupled to the blood flow through $(\Omega _\text {f})_t$ by kinematic and traction compatibility conditions at the fluid–structure interface $(\Gamma _\text {I})_t$. The blood flow within $(\Omega _\text {f})_t$ is assumed to be incompressible and Newtonian. The subscript *t* may be omitted in some formulas below when there is no risk of confusion. This coupled partial differential equation (PDE) system can be expressed in the weak form as: Find a fluid velocity $\textbf{u}_\text {f}\in \mathcal {S}_u$, fluid pressure $p\in \mathcal {S}_p$, structure displacement $\textbf{y}\in \mathcal {S}_y$, and a fluid–solid interface Lagrange multiplier $\pmb {\lambda }\in \mathcal {S}_\ell$ such that for all $\textbf{w}_\text {f}\in \mathcal {V}_u$, $q\in \mathcal {V}_q$, $\textbf{w}_\text {s}\in \mathcal {V}_y$, and $\delta \pmb {\lambda }\in \mathcal {V}_\ell$,

1 $$\begin{aligned} B_\text {f}(&\{\textbf{w}_\text {f},q\},\{\textbf{u}_\text {f},p\})-F_\text {f}(\{\textbf{w}_\text {f},q\}) + B_\text {s}(\textbf{w}_\text {s},\textbf{y})-F_\text {s}(\textbf{w}_\text {s}) \nonumber \\&+\int _{\Gamma _\text {I}}(\textbf{w}_\text {f}-\textbf{w}_\text {s})\cdot \pmb {\lambda }~\text {d}\Gamma + \int _{\Gamma _\text {I}}\delta \pmb {\lambda }\cdot (\textbf{u}_\text {f}-\textbf{u}_\text {s})~\text {d}\Gamma \nonumber \\&+ \int _{\Gamma _\text {I}}(\textbf{w}_\text {f}-\textbf{w}_\text {s})\cdot \beta (\textbf{u}_\text {f}-\textbf{u}_\text {s})~\text {d}\Gamma = 0, \end{aligned}$$

where $\mathcal {S}_{(\cdot )}$ and $\mathcal {V}_{(\cdot )}$ are trial solution and test function spaces, $B_\text {f}$, $B_\text {s}$, $F_\text {f}$, and $F_\text {s}$ are variational forms defining the fluid and structure subproblems, $\textbf{u}_\text {s}$ is the material time derivative of $\textbf{y}$, and $\beta$ is a penalty parameter. The terms integrated over $\Gamma _\text {I}$ enforce the fluid–structure coupling conditions on the fluid–structure interface. The presence of these terms facilitates the development of certain numerical schemes based on the “augmented Lagrangian” concept, as elaborated in Bazilevs et al. [39]. The forms defining the fluid and structure subproblems are detailed in the following sections.

### Structural formulations

This section summarizes the formulations that are required to model the THV, which is composed of the frame, skirt, and leaflets, and the artery wall. Based on the thickness of the THV geometries relative to the overall dimensions of the system, we model the artery wall as an elastic solid and the valve leaflets and skirt as thin shell structures. The frame is fabricated with long, thin wires that are modeled using elastic beams. The following superscripts “be”, “sh”, and “so” denote the beam, shell, and solid, respectively, where $\mathcal {S}_y = \mathcal {S}_y^\text { be}\times \mathcal {S}_y^\text { sh}\times \mathcal {S}_y^\text { so}$ and $\mathcal {V}_y = \mathcal {V}_y^\text { be}\times \mathcal {V}_y^\text { sh}\times \mathcal {V}_y^\text { so}$ such that $\textbf{y} = \left\{ \textbf{y}^\text {be}, \textbf{y}^\text {sh}, \textbf{y}^\text {so}\right\}$ and $\textbf{w}_\text {s} = \left\{ \textbf{w}^\text {be}_\text {s}, \textbf{w}^\text {sh}_\text {s}, \textbf{w}^\text {so}_\text {s}\right\}$. We can then write

2 $$\begin{aligned} B_\text {s}(\textbf{w}_\text {s},\textbf{y})&= B^\text {be}_\text {s}(\textbf{w}^\text {be}_\text {s},\textbf{y}^\text {be}) + B^\text {sh}_\text {s}(\textbf{w}^\text {sh}_\text {s},\textbf{y}^\text {sh})\nonumber \\&~~+ B^\text {so}_\text {s}(\textbf{w}^\text {so}_\text {s},\textbf{y}^\text {so})\text { ,} \end{aligned}$$

and

3 $$\begin{aligned} F_\text {s}(\textbf{w}_\text {s}) = F^\text {be}_\text {s}(\textbf{w}^\text {be}_\text {s}) + F^\text {sh}_\text {s}(\textbf{w}^\text {sh}_\text {s}) + F^\text {so}_\text {s}(\textbf{w}^\text {so}_\text {s})\text { .} \end{aligned}$$

The individual structural formulations for each THV component and the formulations that account for the interaction between components are discussed in the following section.

#### Bernoulli beam formulations for the frame

The stent is fabricated with thin wires and is modeled using an extension of the isogeometric Bernoulli beam. Bauer et al. [40] originally proposed the formulation for geometrically nonlinear spatial curved beams using Bernoulli kinematics. This section summarizes an extension of this beam formulation, developed by Wu et al. [25], that accounts for more complex beam geometries.

In this section, Latin indices take on values $\{1,2,3\}$, and Greek indices take values $\{2,3\}$. The beam formulation begins by defining the continuum of the beam using a centerline and a moving local coordinate system. This coordinate system is used to describe the orientation of the cross sections. The position vector of the continuum of the beam, denoted as $\textbf{x}$ is defined as,

4 $$\begin{aligned} \textbf{x}(\xi ^1,\xi ^2,\xi ^3)=\textbf{r}(\xi ^1)+\xi ^2\,\textbf{a}_2(\xi ^1) +\xi ^3\,\textbf{a}_3(\xi ^1) \text { ,} \end{aligned}$$

where $\textbf{r}$ is the position vector of the centerline, $\xi ^1$ is the contravariant coordinate of the centerline, $\xi ^2$ and $\xi ^3$ are coordinates of the cross-sectional profile, and $\textbf{a}_\alpha$ are unit base vectors tangent to the cross-section and orthogonal to the tangent vector of the centerline denoted as $\textbf{a}_1 = \textbf{r}_{,1}$. The vectors $\textbf{a}_i$ directly define the moving local coordinate system for both the deformed and undeformed configurations. Variables of the latter are denoted by $\mathring{(\cdot )}$.

Bernoulli beam theory assumes the cross sections remain orthogonal to the centerline and the cross-sectional dimensions remain constant throughout deformation. The two components, $\mathring{\textbf{a}}_\alpha$ of the moving local basis at every location *i* along the beam centerline in the undeformed configuration are obtained as,

5 $$\begin{aligned} (\mathring{\textbf{a}}_\alpha )_i = \textbf{R}\left( (\hat{\mathring{{\textbf{a}}}}_1)_i, \mathring{\theta }_i \right) \mathbf {\Lambda } \left( \textbf{A}_1,(\hat{ \mathring{\textbf{a}}}_1)_i \right) \textbf{A}_{\alpha }\text { ,} \end{aligned}$$

$\text {for } i = 1,...,n$, where the three unit vectors $\textbf{A}_{1}$ and $\textbf{A}_{\alpha }$ define a global reference coordinate system, $\mathbf {\Lambda }$ is a mapping operator that maps one vector to the other, $\textbf{R}$ is the rotation operator, $\hat{(\cdot )}$ denotes the normalized vector, and *n* denote the number of points along the centerline, respectively. The mapping and rotation operators used are described in full detail in Bauer et al. [40] and derived based on the Euler–Rodrigues formula specialized to the current problem.

The same two steps of mapping and rotation can then be applied to describe the alignment of the moving local coordinate system from the undeformed configuration to the deformed configuration as follows:

6 $$\begin{aligned} \textbf{a}_\alpha = \textbf{R}(\hat{\textbf{a}}_1, \theta )\mathbf {\Lambda }( \hat{\mathring{\textbf{a}}}_1, \hat{\textbf{a}}_1 )\mathring{\textbf{a}}_\alpha \text { ,} \end{aligned}$$

where $\theta$ is a rotational degree of freedom.

Wu et al. [25] found that the definition of the local coordinate basis described in Eq. (5) is effective; however, for complex structures, the formulation showed some limitations. The extension proposed in Wu et al. [25] is summarized here. Let $(\textbf{a}_{\alpha }')_1 = \mathbf {\Lambda }(\textbf{A}_1,(\hat{ \mathring{\textbf{a}}}_1)_1)\textbf{A}_\alpha$. Then we define,

7 $$\begin{aligned} (\textbf{a}_{\alpha }')_i = \mathbf {\Lambda }((\hat{\textbf{a}}_1)_{i-1}, (\hat{\textbf{a}}_1)_i) (\textbf{a}_\alpha ')_{i-1}\text { ,} \end{aligned}$$

$\text {for } i = 2,...,n$, where *n* is the number of points along the centerline and $(\textbf{a}_{\alpha }')_i$ denotes the vectors $\textbf{a}_{\alpha }'$ at the $i^{\text {th}}$ location. In this work, each $(\textbf{a}_\alpha ')_i$ is evaluated on the quadrature points. We then combine this step with the rotation step to obtain the final formulation defined at every location along the centerline as,

8 $$\begin{aligned} (\mathring{\textbf{a}}_{\alpha })_i = \textbf{R}((\hat{\mathring{\textbf{a}}}_1)_i,\mathring{\theta _i})(\textbf{a}_\alpha ')_i \text { .} \end{aligned}$$

This method helps achieve the desired complex geometry by avoiding twisting at locations where the tangents of the centerline change significantly.

The weak form of the beam subproblem can then be defined as

9 $$\begin{aligned} B^\text {be}_\text {s}(\textbf{w}_\text {s},\textbf{y}) - F^\text {be}_\text {s}(\textbf{w}_\text {s}) =&\int _{(\mathcal {L}^\text {be})_0}\textbf{w}_\text {s}\cdot \rho ^\text {be}_\text {s} A\left. \frac{\partial ^2\textbf{y}}{\partial t^2}\right| _{\mathring{\textbf{x}}}\text {d}\mathcal {L} \nonumber \\&+ \int _{(\mathcal {L}^\text {be})_0}\int _{A}\delta \textbf{E}:\textbf{S}~\text {d}A\text {d}\mathcal {L} \nonumber \\&-\int _{(\mathcal {L}^\text {be})_0}\textbf{w}_\text {s}\cdot \rho ^\text {be}_\text {s} A\textbf{f}_\text {s} ~\text {d}\mathcal {L} \nonumber \\ {}&-\int _{(\mathcal {L}^\text {be})_t}\textbf{w}_\text {s}\cdot \textbf{h}_\text {s}^{\text {net}} ~\text {d}\mathcal {L} \text { ,} \end{aligned}$$

where $(\mathcal {L}^\text {be})_t$ and $(\mathcal {L}^\text {be})_0$ are the centerline of the beam in the deformed and undeformed configurations, respectively, $\textbf{y}$ is the displacement of the centerline, $\partial /\partial t |_{\mathring{\textbf{x}}}$ is the time derivative holding material coordinates $\mathring{\textbf{x}}$ fixed, $\rho ^\text {be}_\text {s}$ is the beam density, $\textbf{S}$ is the second Piola–Kirchhoff stress, $\delta \textbf{E}$ is the variation of the Green–Lagrange strain corresponding to a displacement variation $\textbf{w}_\text {s}$, $\textbf{f}_\text {s}$ is a prescribed body force, $\textbf{h}^\text {net}$ is the total traction from two sides of the beam, and *A* is the cross-sectional area of the beam.

In this work, the stent is modeled using this extension for the IGA Bernoulli beam. Once the centerline curves are defined as discussed in Sect. 2.1, the cross-sectional profiles must also be described. These profiles are defined over the entire NURBS curve using two local unit vectors, $\textbf{v}_2$, and, $\textbf{v}_3$. In this work, the following approach is proposed to determine the orientation of these orthogonal base vectors that define the cross-sectional profiles. Let $\textbf{v}_1$ be the local unit vector that defines the tangent of the curve. Then, define the local unit vector, $\textbf{v}_2$, to be perpendicular to $\textbf{v}_1$ and the cylindrical unit vector,

10 $$\begin{aligned} \hat{\pmb {\phi }}_c = \begin{pmatrix} -\sin {\phi _c} \\ \cos {\phi _c} \\ 0 \end{pmatrix}\text {,} \end{aligned}$$

and $\textbf{v}_3$ to be perpendicular to $\textbf{v}_1$ and $\textbf{v}_2$. Hence, these two unit vectors representing the cross-sectional profile can be defined as

11 $$\begin{aligned} \textbf{v}_2&= \textbf{v}_1\times \hat{\pmb {\phi }}_c \end{aligned}$$

12 $$\begin{aligned} \textbf{v}_3&= \textbf{v}_1\times \textbf{v}_2 \text {.} \end{aligned}$$

In this work, we define a rectangular cross-section with a constant width of 0.54 mm along $\textbf{v}_2$ and a constant width of 0.21 mm along $\textbf{v}_3$ based on the information in Hopf [41]. For the formulation of the Bernoulli beam, we choose $\mathbf {\mathring{a}}_2$ and $\mathbf {\mathring{a}}_3$ to be $\textbf{v}_2$ and $\textbf{v}_3$, respectively. The final stent geometry can be constructed from the curves defined in Sect. 2.1 and the cross-sectional profiles.

The beam structures are discretized using IGA and the Galerkin method. The beam centerlines are defined using cubic NURBS curves and at least $C^1$-continuous NURBS patches to represent the full beam geometry. These basis functions are utilized for the discretization to provide the needed *C*^1^-continuity for the Bernoulli beam formulation.

#### Thin shell formulations for the leaflets and skirt

The leaflets and skirt are thin tissue structures and are modeled as hyperelastic isogeometric Kirchhoff–Love shells [42]. The Kirchhoff–Love hypothesis of straight and normal cross-sections implies that a point $\textbf{x}$ in the shell continuum can be described by a point $\textbf{r}$ on the midsurface and a vector $\textbf{a}_3$ normal to the midsurface: $\textbf{x}(\xi ^1,\xi ^2,\xi ^3)=\textbf{r}(\xi ^1,\xi ^2)+\xi ^3\,\textbf{a}_3(\xi ^1,\xi ^2)$, where $\xi ^1,\xi ^2$ are the contravariant coordinates of the midsurface, $\xi ^3 \in [-H/2,H/2]$ is the through-thickness coordinate, and $H$ is the shell thickness.

 The covariant base vectors and metric coefficients of the convective curvilinear coordinate system are defined by $\textbf{g}_{i}=\textbf{x}_{,i}$ and $g_{ij}=\textbf{g}_{i}\cdot \textbf{g}_{j}$, respectively, where $(\cdot )_{,i}=\partial (\cdot )/\partial \xi ^i$. For the shell formulation, we adopt the convention that Latin indices take on values $\{1,2,3\}$, and Greek indices take on values $\{1,2\}$ to denote the in-plane components. The contravariant base vectors $\textbf{g}^{i}$ are defined by $\textbf{g}^{i}\cdot \textbf{g}_{j}=\delta ^i_j$ and contravariant metric coefficients are given by $[g^{ij}]=[g_{ij}]^{-1}$. For the Kirchhoff–Love shell theory, both normal and transverse shear strains are neglected; only the in-plane strain components are considered. The theory assumes a linear strain distribution through the thickness and defines $g_{\alpha \beta } = a_{\alpha \beta } -2\, \xi ^3b_{\alpha \beta }$, where $a_{\alpha \beta }=\textbf{a}_{\alpha }\cdot \textbf{a}_{\beta },$ $b_{\alpha \beta }=\textbf{a}_{\alpha ,\beta }\cdot \textbf{a}_{3},$ $\textbf{a}_{\alpha }=\textbf{r}_{,\alpha },$ $\textbf{a}_3 = (\textbf{a}_1\times \textbf{a}_2)/||\textbf{a}_1\times \textbf{a}_2||$, and $\Vert \cdot \Vert$ is the Euclidean norm. These definitions hold for both deformed and undeformed configurations where variables of the latter are indicated by $\mathring{(\cdot )}$﻿. The Jacobian determinant of the structure’s motion is $J=\sqrt{\vert g_{ij}\vert /\vert \mathring{g}_{ij}\vert }$ and the in-plane Jacobian determinant is $J_o=\sqrt{\vert g_{\alpha \beta }\vert /\vert \mathring{g}_{\alpha \beta } \vert }$.

The weak form of the shell structural formulation is defined as

13 $$\begin{aligned} B^\text {sh}_\text {s}(\textbf{w}_\text {s},\textbf{y}) - F^\text {sh}_\text {s}(\textbf{w}_\text {s})&=\int _{(\mathcal {S}^\text { sh})_0}\textbf{w}_\text {s}\cdot \rho ^\text {sh}_\text {s} H\left. \frac{\partial ^2\textbf{y}}{\partial t^2}\right| _{\mathring{\textbf{x}}}\text {d}\mathcal {S} \nonumber \\&~~+\int _{(\mathcal {S}^\text { sh})_0}\int _{\frac{-H}{2}}^{\frac{H}{2}}\delta \textbf{E}:\textbf{S}~\text {d}\xi ^3\text {d}\mathcal {S} \nonumber \\&~~-\int _{(\mathcal {S}^\text { sh})_0}\textbf{w}_\text {s}\cdot \rho ^\text {sh}_\text {s} H\textbf{f}_\text {s} ~\text {d}\mathcal {S} \nonumber \\&~~- \int _{(\mathcal {S}^\text { sh})_t}\textbf{w}_\text {s}\cdot \textbf{h}_\text {s}^{\text {net}} ~\text {d}\mathcal {S} \text { ,} \end{aligned}$$

where $(\mathcal {S}^\text { sh})_0$ and $(\mathcal {S}^\text { sh})_t$ are the shell midsurfaces in the reference and deformed configurations, $\textbf{y}$ is the midsurface displacement, $\rho ^\text {sh}_\text {s}$ is the shell density, $\textbf{S}$ is the second Piola–Kirchhoff stress tensor obtained from a hyperelastic strain energy density functional $\psi$: $\textbf{S} =\partial _{\textbf{E}}\psi$, $\textbf{E}= \frac{1}{2}(\textbf{C}-\textbf{I})$ is the Green–Lagrange strain tensor, $\textbf{C}$ is the right Cauchy–Green deformation tensor, $\textbf{I}$ is the identity tensor, $\delta \textbf{E}$ is the variation of $\textbf{E}$ corresponding to displacement variation $\textbf{w}_\text {s}$, $\textbf{f}_\text {s}$ is a prescribed body force, and $\textbf{h}^{\text {net}}_\text {s}$ is the total traction from the two sides of the shell. In this work, the material is assumed to be incompressible. The elastic strain energy functional $\psi _{el}$ is augmented by a constraint term enforcing $J=\sqrt{\text {det}\,\textbf{C}}=1$, via a Lagrange multiplier *p*: $\psi = \psi _{el}-p(J-1)$. Readers are referred to Wu et al. [25] for detailed descriptions of the stress and strain tensors that are used in this work.

#### Artery wall modeling

The artery wall is modeled as a hyperelastic solid subject to damping forces. We define

14 $$\begin{aligned} B^\text {so}_\text {s}(\textbf{w}_\text {s},\textbf{y}) - F^\text {so}_\text {s}(\textbf{w}_\text {s})&= \int _{(\Omega ^\text {so}_\text {s})_0}\textbf{w}_\text {s}\cdot \rho ^\text {so}_\text {s}\left. \frac{\partial ^2\textbf{y}}{\partial t^2}\right| _{\mathring{\textbf{x}}}~\text {d}\Omega \nonumber \\&~~+ \int _{(\Omega ^\text {so}_\text {s})_0} \pmb {\nabla }_{\mathring{\textbf{x}}} \textbf{w}_\text {s} : \textbf{F}(\textbf{S} + \textbf{S}_0)~\text {d}\Omega \nonumber \\&~~-\int _{(\Omega ^\text {so}_\text {s})_0}\textbf{w}_\text {s}\cdot \rho ^\text {so}_\text {s}\textbf{f}_\text {s}~\text {d}\Omega \nonumber \\&~~- \int _{(\Gamma _\text {s}^{\text {so},\text {h}})_t}\textbf{w}_\text {s}\cdot \textbf{h}_\text {s}~\text {d}\Gamma \text { ,} \end{aligned}$$

where $(\Omega ^\text {so}_\text {s})_0$ is the portion of $\Omega _\text {s}$ corresponding to the artery wall in the reference configuration, $\rho ^\text {so}_\text {s}$ is the solid mass density, $\partial (\cdot )/\partial t|_{\mathring{\textbf{x}}}$ is the time derivative holding the material coordinates $\mathring{\textbf{x}}$ fixed, $\pmb {\nabla }_{\mathring{\textbf{x}}}$ is the gradient operator on $(\Omega ^\text {so}_\text {s})_0$, $\textbf{F}$ is the deformation gradient associated with displacement $\textbf{y}$, $\textbf{S}$ is the hyperelastic contribution to the second Piola–Kirchhoff stress tensor, $\textbf{S}_0$ is a prescribed prestress tensor [43, 44], $\textbf{f}_\text {s}$ is a prescribed body force, and $\textbf{h}_\text {s}$ is a prescribed traction on the Neumann boundary $\Gamma _\text {s}^{\text {so},\text {h}}$. In this work, the elastic contribution to the second Piola–Kirchhoff stress in Eq. (14) is derived from a compressible neo-Hookean model with dilatational penalty [43, 45], which is shown to be appropriate for arterial wall modeling in FSI simulations. The additional pressure $\textbf{S}_0$ in Eq. (14) is required because the initial aorta configuration is subject to blood pressure and viscous traction and is therefore not stress-free. We follow the procedure from Xu et al. [29] to determine $\textbf{S}_0$. Additional details can also be found in Wu et al. [25].

#### Structural component interaction

To model the connections between different components of the THV, which would be sutured together in the actual device, the penalty coupling approach proposed by Herrema et al. [46] is adapted for the THV structures. To enforce the shell–shell displacement coupling of the skirt and leaflets at a patch interface $\mathcal {L}_\text {I}^\text {ss}$ between two shell surfaces $\mathcal {S}^\text { sh,A}$ and $\mathcal {S}^\text { sh,B}$, the following displacement penalty term is added to $B^\text {sh}_\text {s}(\textbf{w}_\text {s},\textbf{y})$:

15 $$\begin{aligned} +~\int _{\mathcal {L}_\text {I}^\text {ss}} \alpha _\text {d}^\text {ss} \left( \textbf{w}_\text {s}^\text {sh,A} - \textbf{w}_\text {s}^\text {sh,B}\right) \cdot \left( \textbf{y}^\text {sh,A} - \textbf{y}^\text {sh,B}\right) \text {d}\mathcal {L} \text{, } \end{aligned}$$

where $\textbf{y}^\text {sh,A}$ and $\textbf{y}^\text {sh,B}$ are the displacements on surface patches $\mathcal {S}^\text { sh,A}$ and $\mathcal {S}^\text { sh,B}$, respectively, along the penalty curve $\mathcal {L}_\text {I}^\text {ss}$, $\textbf{w}_\text {s}^\text {sh,A}$ and $\textbf{w}_\text {s}^\text {sh,B}$ are their respective weighting functions, and $\alpha _\text {d}^\text {ss}$ is a variable penalty parameter of sufficiently large magnitude. For shell–beam coupling of the skirt and the frame, the same displacement penalty approach is used, and the following displacement penalty term is added to $B_\text {s}(\textbf{w}_\text {s}, \textbf{y})$:

16 $$\begin{aligned} +~\int _{\mathcal {L}_\text {I}^\text {sb}} \alpha _\text {d}^\text {sb} \left( \textbf{w}_\text {s}^\text {sh} - \textbf{w}_\text {s}^\text {be}\right) \cdot \left( \textbf{y}^\text {sh} - \textbf{y}^\text {be}\right) \text {d}\mathcal {L} \text{, } \end{aligned}$$

where $\alpha _\text {d}^\text {sb}$ is an adjustable penalty parameter, and $\mathcal {L}_\text {I}^\text {sb}$ is a penalty curve along the shell–beam interface. For the shell–beam coupling, in practice, we choose the penalty curve $\mathcal {L}_\text {I}^\text {sb}$ to be $\mathcal {L}^\text {be}$.

To enforce the rotational continuity between two shell surfaces, the penalty approach is also used to maintain the angle formed at the coupled patch interface. The following rotational penalty term [46] is added to $B^\text {sh}_\text {s}(\textbf{w}_\text {s},\textbf{y})$:

17 $$\begin{aligned} +~\int _{\mathcal {L}_\text {I}^\text {ss}}&\alpha _\text {r}^\text {ss}\left( \left( \delta \cos {\phi }-\delta \cos {\mathring{\phi }} \right) \left( \cos {\phi }-\cos {\mathring{\phi }}\right) \right. \nonumber \\ {}&~+ \left. \left( \delta \sin {\phi } - \delta \sin {\mathring{\phi }}\right) \left( \sin {\phi } - \sin {\mathring{\phi }}\right) \right) \text {d}\mathcal {L} \text{, } \end{aligned}$$

where $\mathring{\phi }$ and $\phi$ are the angles between the surfaces before and after deformation, respectively, and $\delta$ denotes the variation. The structure of the THV frame geometry restrains local twisting, so the effect of the beam rotation on the shell is assumed to be negligible, and a rotational penalty is not imposed for the shell–beam coupling.

The penalty parameters for shell–shell coupling are given as

18 $$\begin{aligned} \alpha _\text {d}^\text {ss}&= \alpha ^\text {ss}\frac{E^\text {sh} \, H}{h^\text {sh,I} \, (1-(\nu ^\text {sh})^2)} \text { ,} \end{aligned}$$

19 $$\begin{aligned} \alpha _\text {r}^\text {ss}&= \alpha ^\text {ss}\frac{E^\text {sh} \, H^3}{12 \, h^\text {sh,I} \, (1-(\nu ^\text {sh})^2)} \text { ,} \end{aligned}$$

where $\alpha ^\text {ss}$ is a dimensionless penalty coefficient, $E^\text {sh}$ is some effective material stiffness with units of stress (e.g., the Young’s modulus in a linear isotropic material), $h^\text {sh,I} = (h^\text {sh,A} + h^\text {sh,B})/2$, where $h^\text {sh,A}$ and $h^\text {sh,B}$ are the lengths of the local elements in the direction most parallel to the penalty curve, and $\nu ^\text {sh}$ is the Poisson’s ratio. For shell–beam coupling, the following penalty parameter is employed:

20 $$\begin{aligned} \alpha _\text {d}^\text {sb} = \alpha ^\text {sb}\min \left\{ \frac{E^\text {sh} \, H}{h^\text {sh} \, (1-(\nu ^\text {sh})^2)},\frac{E^\text {be} \sqrt{A}}{h^\text {be} \, (1-(\nu ^\text {be})^2)}\right\} \text { ,} \end{aligned}$$

where $\alpha ^\text {sb}$ is a dimensionless penalty coefficient, $h^\text {sh}$ is defined to be an effective shell element length [25], and $h^\text {be}$ is defined to be the local beam element length. The selection of $\alpha _\text {d}^{\text {sb}}$ from the minimum parameter value between coupled materials produces a sufficiently high penalty value that is not excessive for the lower stiffness material. The penalty parameter is also high enough to ensure constraint satisfaction without creating excessive ill-conditioning.

The THV presents multiple contact problems, including leaflet-to-leaflet, leaflet-to-frame, and frame-to-artery wall contact that can occur during crimping, deployment, and during the cardiac cycle. To model the multiple contact problems, a nonlocal contact formulation with a linear contact kernel is imposed [47]. For contact between two points, $\textbf{x}^\text {A}$ and $\textbf{x}^{\text {B}}$ in a single body with reference configuration $\Omega _0$, the following contact term is added to $B^\text {sh}_\text {s}(\textbf{w}_\text {s},\textbf{y})$:

21 $$\begin{aligned} +\int _{\Omega _0\backslash B_R(\mathring{\textbf{x}}^\text {A})} \int _{\Omega _0 } \delta \textbf{r}^\text {AB}\cdot \phi '_\text {c}(r^\text {AB}) \frac{\textbf{r}^\text {AB}}{r^\text {AB}} \text {d} \mathring{\textbf{x}}^\text {A} \text {d} \mathring{\textbf{x}}^\text {B} \text { ,} \end{aligned}$$

where $B_R(\mathring{\textbf{x}}^\text {A})$ is the Euclidean ball of radius R centered at $\mathring{\textbf{x}}^\text {A}$ in $\Omega _0$, $\textbf{r}^\text {AB} = \textbf{x}^\text {B}-\textbf{x}^\text {A}$, $r^\text {AB} = \Vert \textbf{r}^\text {AB}\Vert$, and $\phi '_\text {c}(r^\text {AB})$ is the linear contact kernel defined as,

22 $$\begin{aligned} \phi '_\text {c}(r^\text {AB}) = -\max (k_\text {c}(r_{\text {max}} -r^\text {AB} ) ,0) \text{, } \end{aligned}$$

where $k_\text {c}$ is a penalty parameter that needs to be sufficiently large and $r_{\text {max}}$ is a cutoff distance within which the contact force is active.

The role of friction is also introduced in the THV system as the deployed device expands and anchors to the aortic wall. In order to model this effect, Wu et al. [25] proposed a simple static friction model to estimate the friction force. The friction model utilizes the following penalty term in locations where contact occurs,

23 $$\begin{aligned} +\int _{\Omega _0^\text {be}}\int _{\Omega _0^\text {so}}\mathcal {F}~ \text {d} \mathring{\textbf{x}}^\text {so} \text {d} \mathring{\textbf{x}}^\text {be} \text { , for } \Vert {\textbf {r}} \Vert < r_\text {max}\text { ,} \end{aligned}$$

where

24 $$\begin{aligned} \mathcal {F} = \delta (\textbf{r})_{\tau }\cdot \alpha _\text {f}~\vert \phi '_\text {c}(\Vert \textbf{r}\Vert )\vert \left( \Vert (\textbf{r})_{\tau }\Vert - \Vert (\textbf{r}_\text {d})_{\tau } \Vert \right) \textstyle \frac{(\textbf{r})_{\tau } }{ \Vert (\textbf{r})_{\tau } \Vert }\text { ,} \end{aligned}$$

$\Omega _0^\text {be}$ and $\Omega _0^\text {so}$ are the reference configurations of the beam and solid, respectively, $\mathring{\textbf{x}}^\text {be}\in \Omega _0^\text {be}$, $\mathring{\textbf{x}}^\text {so}\in \Omega _0^\text {so}$, subscript “d” indicates the deployed configuration, subscript “$\tau$” indicates the tangential component, $\textbf{r} = \textbf{x}^\text {be}-\textbf{x}^\text {so}$, $\textbf{r}_{\text {d}} = \textbf{x}^\text {be}_{\text {d}}-\textbf{x}^\text {so}_{\text {d}}$,

25 $$\begin{aligned} (\textbf{r})_{\tau }&= \textbf{r} - (\textbf{r}\cdot \textbf{n})\textbf{n} \text { ,} \end{aligned}$$

26 $$\begin{aligned} (\textbf{r}_\text {d})_{\tau }&= \textbf{r}_\text {d} - (\textbf{r}_\text {d}\cdot \textbf{n}_\text {d})\textbf{n}_\text {d} \text { ,} \end{aligned}$$

$\textbf{n}$ and $\textbf{n}_\text {d}$ are the outward normal vectors on the artery wall in the current and deployed configurations, respectively, and $\alpha _\text {f}$ is a penalty parameter that is set to $\alpha _\text {f} = 10^{11}$ in this work.

### Fluid formulation

The blood flow in a deforming artery is governed by the Navier–Stokes equations of incompressible flows posed on a moving domain. The fluid subproblem in (1) is given in the arbitrary Lagrangian–Eulerian (ALE) description [48] as follows:

27 $$\begin{aligned}&B_\text {f}(\{\textbf{w}_\text {f},q\},\{\mathbf {u_\text {f}},p\}) - F_\text {f}(\{\textbf{w}_\text {f},q\}) \nonumber \\&~~=\int _{(\Omega _\text {f})_t}\textbf{w}_\text {f}\cdot \rho _\text {f}\left( \left. \frac{\partial \mathbf {u_\text {f}}}{\partial t}\right| _{\hat{\textbf{x}}}+\left( \textbf{u}_\text {f}-\hat{\textbf{u}}\right) \cdot \pmb {\nabla } \textbf{u}_\text {f}\right) ~\text {d}\Omega \nonumber \\ {}&~~~~+ \int _{(\Omega _\text {f})_t}\pmb {\varepsilon }(\textbf{w}_\text {f}):\pmb {\sigma }~\text {d}\Omega + \int _{(\Omega _\text {f})_t}q\pmb {\nabla } \cdot \mathbf {u_\text {f}}~\text {d}\Omega \nonumber \\ {}&~~~~- \gamma \int _{(\Gamma _\text {f}^\text {h})_t}\textbf{w}_\text {f}\cdot \rho _\text {f}\{\left( \mathbf {u_\text {f}}-\hat{\textbf{u}}\right) \cdot \textbf{n}_\text {f}\}\_~\textbf{u}_\text {f}~\text {d}\Gamma \nonumber \\&~~~~-\int _{(\Omega _\text {f})_t}\textbf{w}_\text {f}\cdot \rho _\text {f}\textbf{f}_\text {f}~\text {d}\Omega - \int _{(\Gamma _\text {f}^\text {h})_t}\textbf{w}_\text {f}\cdot \textbf{h}_\text {f}~\text {d}\Gamma , \end{aligned}$$

where $\rho _\text {f}$ is the fluid mass density, $\partial (\cdot )/\partial t|_{\hat{\textbf{x}}}$ is the time derivative taken with respect to the fixed coordinates $\hat{\textbf{x}}$ of the spatial configuration, $\hat{\textbf{u}}$ is the (arbitrary) velocity with which the fluid subproblem domain ${(\Omega _\text {f})_t}$ deforms, $\pmb {\nabla }$ is the gradient taken with respect to the spatial coordinate $\textbf{x}$ of the current configuration, $\pmb {\varepsilon }(\cdot )$ is the symmetric gradient operator given by $\pmb {\varepsilon }(\textbf{w}) = \frac{1}{2}(\pmb {\nabla } \textbf{w}+\pmb {\nabla } \textbf{w}^\text {T})$, $\pmb {\sigma } = -p\textbf{I} + 2 \mu _\text {f} \, \pmb {\varepsilon }(\mathbf {u_\text {f}})$ is the fluid Cauchy stress, $\mu _\text {f}$ is the dynamic viscosity, the $\gamma$ $(\ge 0)$ term, referred to as backflow stabilization [49], improves the well-posedness of the problem when there is significant inflow through the Neumann boundary $\Gamma _\text {f}^\text {h}$, $\{\cdot \}\_$ isolates the negative part of its argument, $\textbf{n}_\text {f}$ is the outward-facing normal vector to the fluid domain, $\textbf{f}_\text {f}$ is a prescribed body force, and $\textbf{h}_\text {f}$ is a prescribed flux on $\Gamma _\text {f}^\text {h}$. This flux is defined as a traction on the outflow portions of the boundary (where $\left( \mathbf {u_\text {f}}-\hat{\textbf{u}}\right) \cdot \textbf{n}_\text {f} > 0$) and some $\gamma$-dependent combination of traction and advective flux on the inflow portion of the boundary [50].

### Immersogeometric discretization of the FSI model

#### Fluid and structure subproblems

The ALE Navier–Stokes subproblem is discretized using a combination of nonuniform rational B-spline (NURBS)-based IGA and the variational multiscale (VMS) approach [51–55], with some modifications to the stabilization parameters to improve mass conservation, as detailed in Kamensky et al. [33, Section 4.4]. The ALE–VMS formulation may be interpreted both as a stabilized formulation and a large-eddy simulation turbulence model. The methodology applies equally well to laminar and turbulent flows and is thus attractive for the current application where the nature of the flow solution is not known a priori. The VMS formulation maintains stability over broad classes of velocity and pressure discrete spaces and ensures that solutions are not restricted to special inf–sup-stable combinations. For computations in this paper, an “equal order” discretization scheme is used—the same scalar discrete space is used to represent the pressure and each Cartesian component of the fluid velocity. On the fluid mechanics domain interior, the mesh velocity $\hat{\textbf{u}}$ is obtained by solving a fictitious linear elastostatic problem subject to the displacement boundary conditions coming from the motion of the fluid–solid interface from each time step to the next [56–60]. The solid, shell, and beam structures are discretized using IGA and the Galerkin method. At least $C^1$-continuous NURBS patches are used to represent the geometries. NURBS are also used as basis functions for discretization because they can easily provide the required *C*^1^-continuity between elements for the Kirchhoff–Love shell and Bernoulli beam formulations.

#### Discretization of the fluid–structure kinematic constraint

The artery wall and blood flow domains are chosen to have conforming discretizations at the fluid–solid interface. This automatically satisfies the kinematic and traction compatibility conditions due to the fact that $\textbf{u}_\text {f} = \textbf{u}_\text {s}$ and $\textbf{w}_\text {f}=\textbf{w}_\text {s}$ at the interface, causing the Lagrange multiplier and penalty terms in Eq. (1) to be zeros. This is not the case at the fluid–shell interface.  The fluid–shell coupling utilizes the Lagrange multiplier and penalty methods to enforce the kinematic constraints. To take advantage of the weak imposition of no-slip conditions at the fluid–shell interface [39, 61], the tangential component of the Lagrange multiplier $\pmb {\lambda }$ is formally eliminated, leaving only the penalty method to weakly enforce the no-slip condition. However, due to the presence of large pressure gradients across the leaflets, the normal component of the Lagrange multiplier $\lambda = \pmb {\lambda } \cdot \textbf{n}^\text {sh}_\text {s}$, where $\textbf{n}^\text {sh}_\text {s}$ is the normal vector of the shell midsurface, is retained at the fluid–shell interface in order to strengthen the enforcement of the no-penetration condition. For fluid–beam coupling, only the penalty is used to weakly enforce the kinematic constraints due to the fact that the interaction force between the beam and the blood flow is not high.

The discrete variational equation for this FSI system is summarized as follows: Find $\textbf{u}^h_\text {f}\in \mathcal {S}_u^{\text{ }h}$, $p^h\in \mathcal {S}_p^{\text{ }h}$, $\textbf{y}^h\in \mathcal {S}_y^{\text{ }h}$, and $\lambda \in \mathcal {S}_\ell ^{\text{ }h}$ such that for all $\textbf{w}_\text {f}^h\in \mathcal {V}_u^{\text{ }h}$, $q^h\in \mathcal {V}_q^{\text{ }h}$, $\textbf{w}_\text {s}^h\in \mathcal {V}_y^{\text{ }h}$, and $\delta \lambda \in \mathcal {V}_\ell ^{\text{ }h}$,

28 $$\begin{aligned}&B_\text {f}^{\text {VMS}}\left( \{\textbf{w}^h_\mathtt{{f}},q^h\},\{\textbf{u}^h_\text {f},p^h\}\right) -F_\text {f}^{\text {VMS}}\left( \{\textbf{w}^h_\text {f},q^h\}\right) \nonumber \\&~~+ B_\text {s}\left( \textbf{w}^h_\text {s},\textbf{y}^h\right) -F_\text {s}\left( \textbf{w}^h_\text {s}\right) \nonumber \\&~~+ \int _{\mathcal {S}^\text { sh}}\left( \textbf{w}_\text {f}^h-\textbf{w}_\text {s}^{\text {sh},h}\right) \cdot \lambda \textbf{n}_\text {s}^\text {sh}~\text {d}\mathcal {S} \nonumber \\&~~+\int _{\mathcal {S}^\text { sh}}\delta \lambda \left( \left( \textbf{u}_\text {f}^h-\textbf{u}_\text {s}^{\text {sh},h}\right) \cdot \textbf{n}_\text {s}^\text {sh} - \frac{r}{\beta ^\text {sh}_{\text {NOR}}}\lambda \right) ~\text {d}\mathcal {S} \nonumber \\&~~+ \int _{\mathcal {S}^\text { sh}}\left( \textbf{w}^h_\text {f}-\textbf{w}_\text {s}^{\text {sh},h}\right) \cdot \beta ^\text {sh}_{\text {NOR}}\left( \left( \textbf{u}_\text {f}^h-\textbf{u}_\text {s}^{\text {sh},h}\right) \cdot \textbf{n}_\text {s}^\text {sh}\right) \textbf{n}_\text {s}^\text {sh}~\text {d}\mathcal {S} \nonumber \\&~~+ \int _{\mathcal {S}^\text { sh}} \left( \textbf{w}_\text {f}^h-\textbf{w}_\text {s}^{\text {sh},h}\right) \cdot \beta ^\text {sh}_{\text {TAN}}\left( \left( \textbf{u}_\text {f}^h-\textbf{u}_\text {s}^{\text {sh},h}\right) \right. \nonumber \\&\hspace{3.67cm}- \left. \left( \left( \textbf{u}_\text {f}^h-\textbf{u}_\text {s}^{\text {sh},h}\right) \cdot \textbf{n}_\text {s}^\text {sh}\right) \textbf{n}_\text {s}^\text {sh}\right) ~\text {d}\mathcal {S} \nonumber \\&~~+ \int _{\mathcal {L}^\text {be}}\left( \textbf{w}_\text {f}^h-\textbf{w}_\text {s}^{\text {be},h}\right) \cdot \beta ^\text {be}\left( \textbf{u}_\text {f}^h-\textbf{u}_\text {s}^{\text {be},h}\right) ~\text {d}\mathcal {L} =~ 0 \text { ,} \end{aligned}$$

$F_{\text {f}}^{\text {VMS}}$ are the VMS discretizations of $B_\text {f}$ and $F_{\text {f}}$, respectively, the superscript *h* denotes the corresponding variable in the discrete space, $\beta ^\text {sh}$’s and $\beta ^\text {be}$ are penalty parameters for fluid–shell and fluid–beam interfaces, respectively, and $r \ge 0$ is a dimensionless constant, typically $\ll 1$. The term associated with *r* is introduced to regularize the no-penetration constraint in order to circumvent the inf–sup condition (see [62] for details) and it can be viewed as a degenerate case of strongly-consistent Barbosa–Hughes stabilization [63]. It is important to note here that in Eq. (1), the fluid–structure coupling terms are integrated over $\Gamma _\text {I}$. These  terms are separated and $\Gamma _\text {I}$ is chosen as  $\mathcal {L}^\text {be}$ for the fluid–beam term and $\mathcal {S}^\text { sh}$ for the fluid–shell terms.

### Time integration and solution strategies

To solve the FSI equation system, we utilize a two-step, semi-implicit time integration procedure. The first step is to solve implicitly for the fluid, solid structure, mesh displacement, shell structure, and beam structure unknowns at the $n+1$ time level while holding the Lagrange multiplier $\lambda$ fixed at its current value. The unknowns are solved using a combination of quasi-direct and block-iterative coupling strategies [64, 65]. The second step is to update the Lagrange multiplier by adding the normal component of penalty forces from the fluid and structure solutions in the first step. A detailed analysis of stability and accuracy of this methodology can be found in Kamensky et al. [66] and Yu et al. [67].

## Simulation results and discussion

### Simulation setup

The aorta geometry is shown in Fig. 1A. The aortic annulus diameter is 23 mm, and the other dimensions of the aorta geometry are defined in Wu et al. [25]. The computational domain is made up of quadratic trivariate NURBS elements with 88,560 fluid elements comprising the fluid domain and 8640 solid elements comprising the artery wall. The fluid and solid elements share nodes at the interface, making the system $C^0$-continuous. Based on the dimensions of the selected THV-A and THV-B valve configurations, which both have 26 mm valve diameters in the base region, this aortic size is consistent with the recommended selection of the implant dimensions, which should be appropriately oversized compared to the aorta to ensure stable implantation.

For simplicity and to maintain consistent material parameters for both geometric configurations, the same material properties are used for the THV-A and THV-B. The frame is modeled using the St. Venant–Kirchhoff material model with a Young’s modulus of $E = 58$ GPa and Poisson’s ratio of $\nu = 0.33$ [68]. The mass density of the frame is set to 6.45 g/cm$^3$. The tissue properties in this work are selected to approximate porcine pericardial tissue [69].

In this work, the constitutive behavior of the THV leaflets and skirts is modeled using a transversely isotropic (Lee–Sacks) model [70, 71]. The strain energy functional in this model uses a neo-Hookean term to simulate the extracellular matrix and a convex combination of fully-isotropic and transversely-isotropic exponential-type terms to simulate the network of collagen fibers. The model is given by

29 $$\begin{aligned}&\psi _{el}(I_1,I_4) = \frac{c_0}{2}\left( I_1-3\right) \nonumber \\&~~+ \frac{c_1}{2}\left( w e^{c_2(I_1-3)^2} + (1-w) e^{c_3(I_4-1)^2}-1\right) \text { ,} \end{aligned}$$

where $c_0$, $c_1$, $c_2$, and $c_3$ are material parameters, $w\in [0,1]$ determines the strain energy contribution due to anisotropy, $I_1 = \text {tr}\,\textbf{C}$, and $I_4 = \textbf{m}\cdot \textbf{C}\,\textbf{m}$, where $\textbf{m}$ is a unit vector defining the collagen fiber direction in the reference configuration. Based on the structure of the bovine or porcine pericardium that is typically used in THVs, we assume that the collagen fibers lie primarily in the plane of the tissue. For numerical stability, a hybrid material constraint [72] is employed to enforce upper bounds for the $I_1$ and $I_4$ terms in $\psi _{el}$ based on the properties of the system. The material fitting procedure proposed in Wu et al. [71] is used to obtain the parameters for the Lee–Sacks material model based on the $0^\circ$-fiber biaxial mechanical properties of porcine pericardium reported in Caballero et al. [73]. The following parameters are obtained for the leaflet tissue: $c_0 = 117.1375$ kPa, $c_1 = 41.4347$ kPa, $c_2 = 109.7423$, $c_3 = 132.4545$, and $\delta = 0.9883$. In this work, we assume a fiber orientation of $45^\circ$ for the leaflets based on the observed fiber orientation of native porcine valves [74].

The fiber orientation of the skirt is unclear, so the skirt is modeled using the Lee–Sacks isotropic material model for simplicity. The following parameters are obtained for the skirt tissue: $c_0 = 117.1375$﻿ kPa, $c_1 = 60.3991$ kPa, $c_2 = 92.4784$, and $\delta = 1$. The thickness of the skirt and leaflets is set to 0.033 cm [69], and their mass densities are set to 1.0 g/cm$^3$.

For the THV-A geometry analyzed in this work, each section of the skirt is discretized with 220 cubic NURBS elements, each leaflet is discretized with 380 cubic NURBS elements, and each commissural joint is discretized with 24 cubic NURBS elements. Each section of the stent is generated by evaluating the baseline stent curve with two levels of *h*-refinement to obtain 112 cubic elements to define an analysis-suitable curve.

For the THV-B geometry analyzed in this work, each section of the skirt is discretized with 189 cubic NURBS elements, each leaflet is discretized with 216 cubic NURBS elements, and each commissural joint is discretized with 6 cubic NURBS elements. Each section of the stent is generated by evaluating the baseline stent curve with two levels of *h*-refinement to obtain 76 cubic elements to define an analysis-suitable curve.

A value of $\alpha ^\text {ss} = 10^3$, as proposed by Herrema et al. [46], is appropriate for the shell–shell coupling for this application. In this work, $\alpha ^\text {sb} = 10^4$ for shell–beam coupling. To apply this coupling method to the hyperelastic material model of the leaflets and skirt, $E^\text {sh}$ is calculated based on the shear modulus of the neo-Hookean term ($c_0$) and $\nu ^\text {sh}$ is set to 0.5. Additional simulation parameters and constraints for the fluid–structure coupling and the contact problem can be found in Wu et al. [25].

### Crimping and deployment setup and simulation

To demonstrate the implantation process and deployed properties of such devices, each valve configuration is positioned in the aorta through a crimping and deployment process similar to the realistic deployment that a self-expandable TAVR device would undergo. The valve crimping procedure is performed through a structural simulation that does not involve any fluid. A radially inward force that is nonuniform along the vertical length of the frames is applied to ensure that the radius of the valves is sufficiently reduced to fit within the aorta [25].

To obtain the artery wall prestress used in the THV deployment and subsequent FSI simulation, we apply the left ventricular pressure at peak systole ($t =$ 0.25 s) at the inlet and a resistance boundary condition as traction at the outlet. All other boundary conditions are consistent with those explained below. The prestress tensor is then calculated and used as an input in subsequent steps. Additional details can be found in Wu et al. [25].

The valve deployment is performed using FSI with the following simulation properties and setup. The pressure at time $t = 0.6$ s, as shown in Fig. 1A, is applied as a traction boundary condition at the inlet [25]. The same resistance boundary condition for both the deployment setup and simulation is applied at the outlet as the traction, $-(p_0 + RQ)\textbf{n}_\text {f}$, where $p_0=80$ mmHg is a constant physiological pressure level, $R=70$ (dyn s)/cm$^5$ is a resistance constant, *Q* is the volumetric flow rate through the outlet, and $\textbf{n}_\text {f}$ is the outward facing normal of the fluid domain, as shown in Fig. 1A. The fluid density and viscosity are $\rho _\text {f} = 1.0 \text { g}/\text {cm}^3$ and $\mu _\text {f}=3.0\times 10^{-2} \text { g}/(\text {cm } \text {s})$, respectively, which model the physical properties of human blood. The backflow stabilization with $\gamma =0.5$ is applied at both the inlet and outlet. The same fluid properties, resistance boundary condition, and backflow stabilization are applied for all the FSI simulations in this work.

For simplicity, no additional leaflets and tissues that would replicate the effect of the native leaflets being crushed into the aortic sinus region during deployment are simulated in this work. The location of each device is maintained throughout the deployment to situate the leaflets at a relatively realistic location in proximity to the bottom of the aortic sinuses [25].

**Fig. 10:**
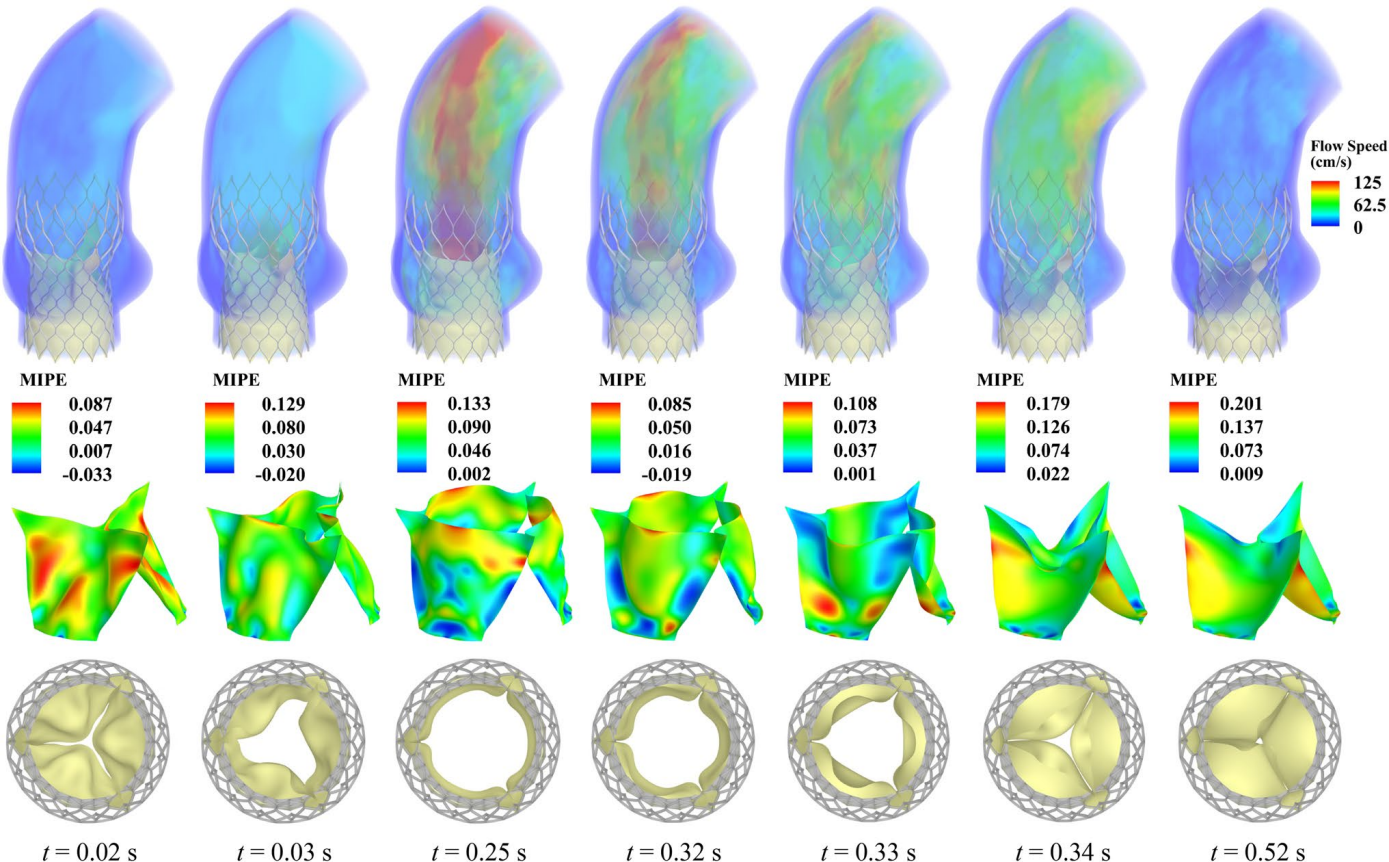
Results for the THV-A valve configuration. The flow results are shown as a volume rendering of the velocity field at several points during a cardiac cycle. The time *t* is synchronized based on the current cycle. The strain map (MIPE) on the leaflets and the top view of the THV are also shown at each time. The strains are evaluated on the aortic side of the leaflets

**Fig. 11:**
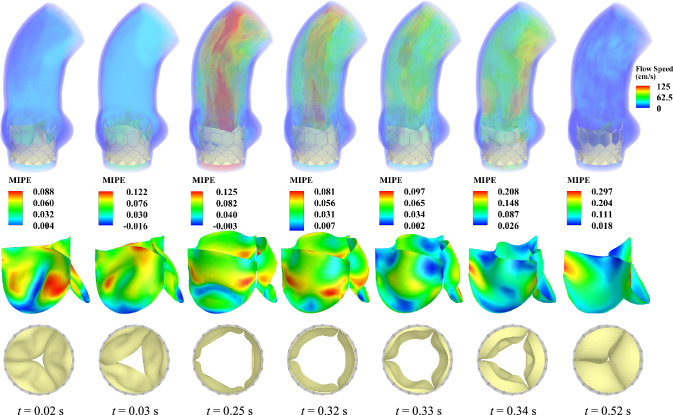
Results for the THV-B valve configuration. The flow results are shown as a volume rendering of the velocity field at several points during a cardiac cycle. The time *t* is synchronized based on the current cycle. The strain map (MIPE) on the leaflets and the top view of the THV are also shown at each time. The strains are evaluated on the aortic side of the leaflets

### FSI setup and simulation throughout the cardiac cycle

To simulate the full cardiac cycle, FSI simulation is performed with a physiologically realistic left ventricular (LV) pressure profile applied as a traction boundary condition on the inlet, as shown in Fig. 1A. The same fluid properties and other boundary conditions from the deployment are carried over to the full FSI simulation of the cardiac cycle.

In an effort to maintain consistency between the deployment and the simulation of the cardiac cycle, the FSI simulation starts at time $t = 0.6$ s using the deployed configuration as the initial condition. For both valve configurations, a single cardiac cycle is 0.86 s, and the time step size is set to $\Delta t = 1.0 \times 10^{-4}$ s and $\Delta t = 5.0 \times 10^{-5}$ s during opening and closing periods of the cardiac cycle, respectively. The time step is decreased during the closing portion of the cycle to accurately capture the hammering effects and challenging contact behavior of the leaflets during the initial closure. Two complete cardiac cycles were simulated for each valve configuration, and the final cycle was selected for the presented results and discussion for this demonstration.

### Results

The following section presents the FSI results for the two valve configurations that are modeled in this work. While these results provide a relatively comprehensive overview of many of the important FSI simulation outputs, they are not exhaustive and are primarily intended to provide a demonstration of how this geometry modeling and analysis framework can be leveraged to identify various factors of interest when investigating different THV geometries.

Figures 10 and 11 correspond to the results for the THV-A and THV-B models, respectively, and include the primary FSI results for these two valves. In these figures, the flow results show the volume rendering of the velocity field at several points during a cardiac cycle. The maximum in-plane principal Green-Lagrange strain (MIPE) is shown on the aortic side of the leaflets. Figures 10 and 11 also show the valve opening area from the top view at the same time instances.

**Fig. 12:**
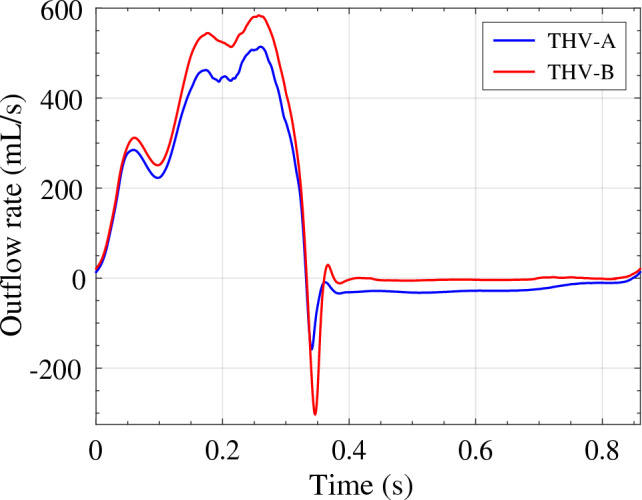
Outflow rate for each valve configuration

**Fig. 13:**
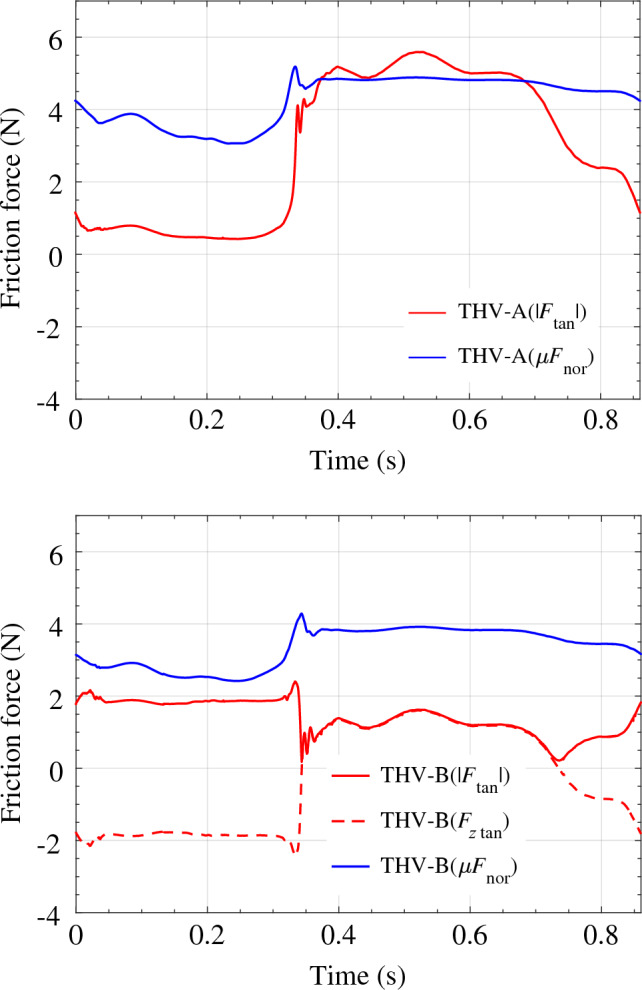
Frictional forces in each valve configuration

In addition to the primary FSI results, two additional quantities of interest are also evaluated. Results for the aortic outflow rate for each valve configuration are computed and shown in Fig. 12, and results for the frictional forces evaluated on each valve are shown in Fig. 13. For each valve, the frictional forces are evaluated using two different methods to assess the relative frictional properties of each configuration. In these results, $|F_{\text {tan}}|$ represents the evaluated magnitude of the frictional force that would be required to oppose the fluid forces exerted on the valve structure throughout the cardiac cycle. For the THV-B, the friction force in the *z*-direction, $F_{z\,\text {tan}}$, is also shown to supplement the magnitude. The other force shown in both plots, $\mu F_{\text {nor}}$, is a calculated friction force that is evaluated from the radial force (i.e., the normal force) of the stent on the aortic wall and multiplied by an assumed coefficient of friction. In this work, a coefficient of friction of $\mu = 0.2$ is selected to roughly approximate the static friction force that could be present for these deployed devices, including the effects of calcium deposits and the native leaflets, which would increase the effective coefficient of friction [75].

### Discussion

In both models shown in Figs. 10 and 11, the three leaflets maintain relatively similar behavior for a particular configuration at any time during the cycle, and the leaflets do not seem to be significantly impeded by the stent. The overall flow behavior is comparable in both cases, with the THV-B valve exhibiting slightly higher flow speeds over a larger region of the aorta that are also reflected by the higher outflow rates in Fig. 12. The high-speed jet in the THV-B case is wider than that of the THV-A, and the overall behavior of the high-speed jet may also correspond to the slightly larger radial dimension of the THV-B (see $P_2$ to $P_8$ in Tables 1 and 2) and the larger orifice area that is observed at the maximum opening time. As the valves close, similar flow behavior is observed, but the THV-B exhibits more stationary flow, which likely indicates better valve closure compared to the THV-A.

There are some strain concentrations observed on the leaflets that are generally localized near the attachment edges and corners of the valve. During the valve closure, both models demonstrated pinwheeling behavior, which is a common phenomenon in bioprosthetic valves due to sizing and closure requirements; however, this effect can also lead to asymmetric fatigue behavior and shortened valve lifetimes [76]. Figures 10 and 11 reveal the expected asymmetric strain distributions along the leaflets due to the pinwheel closure. Both configurations exhibit similar overall strain levels between consistent time steps; however, any regions with high strain concentrations could contribute to additional fatigue in those areas over the valve lifetime. The strain results in both cases also remain consistent with expected values and are comparable to those reported in other relevant literature.

Both models exhibit a large opening area when the leaflets are in the fully open position, indicating a good ejection fraction and minimal obstruction to the blood flow, which should enhance circulation and maximum flow rate. An examination of the valve top views indicates asymmetry during the opening portion of the cardiac cycle for both valves. In the fully open configuration, the THV-B remains somewhat asymmetric, while the THV-A leaflets open into a visually symmetric formation. Comparing the two valves, the THV-A demonstrates a smaller orifice area, and its shape remains more consistent throughout the cardiac cycle. However, it still exhibits asymmetric closure. As noted previously, the smaller orifice area of the THV-A may be related to the larger radial dimension of the THV-B (see $P_2$ to $P_8$ in Tables 1 and 2), but this difference could also be a result of the valve configuration, leaflet curvature, etc.

From the outflow rate results, it is evident that the maximum flow rate for the THV-B is greater than that of the THV-A, which may also correspond to the larger radial dimension of the THV-B and the larger orifice area. The flow velocity of the THV-B reaches a peak value of approximately 583 mL/s between 0.25 and 0.26 s, which may produce more desirable flow characteristics to ensure proper circulation. The magnitude of the minimum flow rate is also smaller for the THV-A with a value of approximately −158 mL/s around 0.34 s, compared to approximately $-304\text { mL/s}$ for the THV-B; however, the THV-B flow rate returns to near zero once the valve is closed. This may indicate that the THV-A geometry could provide more stable flow behavior across the valve but could result in potential leakage if the valve does not seal properly. Since the simulation used the same material and setup for both valves, these contrasting FSI results highlight the differences that are induced primarily due to geometric changes in the device configuration.

As shown in Fig. 13, the valves experience relatively different frictional behavior. In the THV-A case, all the components of the tangential friction force remain positive throughout the cardiac cycle, indicating that the valve would be at risk of migrating downward if the fluid forces during the cycle exceeded the static friction forces. This is not the case for the THV-B configuration, which has a negative component in the *z*-direction during the opening period that would cause the valve to migrate upward if the fluid forces exceeded the required static friction forces during this portion of the cycle. This direction then reverses for a portion of the closure period of the cardiac cycle. In both cases, the valves are most likely to experience migration in situations where the evaluated tangential force of friction exceeds the estimated friction force, $\mu F_{\text {nor}}$. This situation does not occur in the THV-B case based on the specified frictional properties, but the required magnitude of the friction force is relatively high during the opening section of the cycle, so this would be the time when this valve would be at a higher risk of migration in the case of modified frictional properties. In the THV-A case, the required magnitude of the frictional force does exceed the calculated friction during a portion of the closure. While the calculated friction forces only estimate the approximate level of friction that may be present in this scenario, the valve would be most likely to migrate during this portion of the cycle. This situation might also require some additional device treatments to increase the coefficient of friction or sizing changes that would increase the normal forces on the THV.

## Conclusion

This demonstration of the proposed framework highlights the varying outcomes that result from these drastically different valve geometries. In real-life scenarios, each transcatheter valve design possesses distinct material properties, deployment techniques, and valvular structures that introduce even more variation in the performance and overall behavior of different devices. The distinct results comparisons presented in this work emphasize the importance of investigating device sensitivities, including valvular sizing for different aortic diameters, as well as the effectiveness of a parameterized valve modeling framework that enables rapid design generation and exploration. Even for devices that aim to address the same issue of a diseased valve, the vastly different designs and results outputs reflect the significant challenge of non-unique design solutions to a complex, real-world problem.

Through this proposed framework, further investigations of standard valvular sizes and configurations can be examined to improve and optimize valve performance. Using this type of parametric modeling approach, all aspects of these THVs can be consistently updated relative to the other valve dimensions. This key feature could enable efficient future examinations of how changes in diameter, leaflet curvature, or other leaflet parameters impact valve behavior. With systematic adjustments and observations that could directly integrate traditional optimization methods with the proposed computational modeling framework, optimal valve designs for specified design conditions could be efficiently identified and evaluated. The benefits of a computational modeling framework are particularly advantageous in such cases because they avoid more costly experimental design iterations that might only provide incremental improvements in the flow behavior and structural integrity of developed devices. Future improvements to the proposed framework are being considered to streamline and generalize the stent modeling approaches and investigate the presence of native valves, including different calcification levels, to take steps toward accurately simulating patient-specific outcomes. Overall, these streamlined modeling and simulation techniques for transcatheter aortic valve development have the potential to enable more rapid exploration of design options, enhance capabilities for generating valves based on individual patient needs, and improve clinical outcomes.

## Data Availability

The data that support the findings of this study are available from the corresponding author upon reasonable request.
